# Corruption distance and equity strategies in cross-border M&As: The moderating roles of political connections

**DOI:** 10.1371/journal.pone.0325968

**Published:** 2025-06-11

**Authors:** Xiaoyuan Li

**Affiliations:** School of Economics and Management, Sichuan Normal University, Chengdu, China; Ovidius University of Constanta: Universitatea Ovidius din Constanta, ROMANIA

## Abstract

This study explores the impact of corruption distance between home and the host countries on equity strategies in cross-border mergers and acquisitions (M&As). In addition, this study evaluates the moderating roles of two distinctive types of political connections. Analyzing cross-border M&A transactions by Chinese acquirers from 2016 to 2021, this study finds that greater corruption distance significantly increases the equity percentage of acquisitions by Chinese acquirers. This is because a greater corruption distance prompts Chinese acquirers to prefer a higher equity percentage, aimed at mitigating transaction costs and securing control over the target firm. Notably, this study argues that inherent political connections weaken the positive effect of corruption distance on the equity percentage of acquisitions; whereas acquired political connections strengthen its positive effect. In conclusion, this study extends the existing literature of the cross-border equity strategies of enterprises from emerging markets and offers practical insights for Chinese enterprises in managing complex cross-border M&A activities.

## 1. Introduction

Cross-border mergers and acquisitions (M&As) offer unique advantages over greenfield investments, allowing companies to rapidly penetrate foreign markets, expand market share, and assimilate the valuable resources and cutting-edge technology of acquired firms [1]. For this reason, enterprises, particularly those from emerging markets, have embraced cross-border M&As as a critical avenue for international expansion and enhancing their competitive advantage [2–4].

In cross-border M&As, *equity strategy*, defined as the systemic policies that determine the distribution of equity and control in a cross-border M&A deal, should be robust [5–7]. A high equity stake enables the acquirer to exert greater control over the target enterprise; nevertheless, integration risks, largely cultural in nature, as well as those concerning management and operation, should not be overlooked [8]. Whereas, a low equity strategy may place lesser direct costs and risks on the acquirer; however, such a strategy faces other challenges in coordination, and inefficient overseas operations [9]. Therefore, an optimal equity strategy is not merely the cornerstone of successful cross-border M&As but also a critical factor of ongoing operations, post-merger performance, and long-term growth [10].

Relevant literature on cross-border M&As points to a variety of factors influencing equity strategies, including the host country’s economic progress, institutional strength, geographical proximity, and cultural similarities with the home country [11,12]. These exogenous uncertainties carry significant implications for the equity strategies employed by multinational enterprises (MNEs) [8]. Corruption, a key indicator of institutional robustness, represents a proxy for the overall stability of the investment climate, thereby significant affecting the process and performance of cross-border M&As [13]. The implications of corruption for MNEs have been explored in numerous studies with differing perspectives. One line of research suggests that in the face of high corruption levels in the host country, MNEs, wary of inflated operating costs and potential hazards, gravitate towards joint ventures with local partners, viewing these partnerships as a means of tempering the direct effect of corruption and operational risks [14,15]. Contrarily, other studies hypothesize that high host-country corruption might incentivize MNEs to pursue full acquisitions, accelerating internalization efforts to reduce transaction costs arising from information asymmetry and enabling more agile responses to corruption-related challenges through direct target control [16].

Existing research into the effect of corruption on MNEs’ foreign direct investment (FDI) has produced inconsistent conclusions, often attributable to different theoretical frameworks. To offer a more accurate understanding of this relationship, this study proposes the concept of *corruption distance,* which refers to the difference in corruption levels between the acquirer’s home country and the target’s host country [17]. In comparison to focusing on the absolute level of corruption in the host country, “corruption distance” focuses on the comparative relationship between country pairs on bilateral FDI. This concept captures external uncertainties more accurately, thereby improving MNEs’ cross-border M&A transaction equity strategy rationale [5,6].

Moreover, governments significantly affect enterprises in emerging economies during cross-border M&As. Underdeveloped market mechanisms and ambiguous political conditions in these emerging markets are primarily responsible for this governmental influence [18]. Specifically, in relationship-driven emerging economies such as China, political connections between corporations and the government crucially substitute for robust market mechanisms [19,20]. China’s unique political and economic environment highlights the critical role of political connections in corporate decision-making for cross-border M&As. Chinese MNEs can leverage these connections to develop equity strategies balancing domestic institutional voids with international market expectations, thereby reducing geopolitical risks [21]. Consider the acquisition of Volvo Cars by Geely in 2010 as an example involving a Chinese state-owned enterprise acquiring a foreign firm. In this case, political connections were crucial for resolving regulatory barriers and facilitating a smooth transaction. The Chinese government often facilitates such acquisitions through favorable policy support and financial backing [22], demonstrating how vital political connections are for overcoming potential deal-blocking challenges in cross-border M&As [23,24]. This study, therefore, builds upon this context to evaluate how political connections in Chinese firms moderate the relationship between corruption distance and equity strategies in cross-border M&As.

Based on prior studies [20,25], this study distinguishes between two main types of political connections: (1) inherent political connections, primarily demonstrated through state ownership in corporate equity, and (2) acquired political connections, consisting of executives’ memberships in organizations such as the National People’s Congress and the Chinese People’s Political Consultative Conference, or their past employment in government agencies. The literature suggests that these two connection types may produce different implications for cross-border M&As [26]. Specifically, inherent political connections are generally regarded as passive, structural resources. Acquired political connections, however, are considered more active resources that are strategically developed [20,26]. This study hypothesizes, based on these distinctions, that the moderating effects of these two political connection types on the corruption distance–equity strategy relationship in cross-border M&As could differ.

## 2. Theoretical background and hypotheses development

### 2.1. Corruption in cross-border M&As

Institutions represent the “rules of the game” governing interactions among social entities. Institutional theory suggests a host country’s institutional environment significantly shapes firms’ decisions regarding cross-border M&As. Corruption, a key institutional factor, is the focus of this study, analyzing its effects on cross-border M&As. Previous studies demonstrated corruption’s potential to impede economic growth [27], undermine government legitimacy [28], and impact political and social stability [29]. Therefore, corruption in the host country is frequently treated as an additional business expense when analyzing cross-border M&As. Habib and Zurawicki [17] proposed that foreign investors might avoid corruption for ethical reasons. They also suggested investors seek to circumvent it due to the risks, management challenges, and additionally local operational costs. According to Shleifer and Vishny [30] and Aidt [31], corruption in an economy function as a “grabbing hand.” It inflates the costs associated with business activities such as FDI.

Notwithstanding the expectation of these findings, Egger and Winner [32] identified a more nuanced relationship between corruption and FDI. Their analysis indicated a negative correlation between corruption and FDI; however, its influence differs among countries and evolves over time. Similarly, Hakkala et al. [33] distinguished between horizontal and vertical Swedish FDI types. They observed that corruption generally lowers the probability of investing in a nation. However, once the investment decision is accorded, corruption reduces horizontal FDI while having minimal impact on vertical FDI. This observation highlights that horizontal and vertical FDI react differently to host country corruption, indicating FDI should not be treated as a uniform phenomenon.

A contrasting perspective comes from Bjorvatn and Søreide [34] who contended that bribes might enable MNEs to bypass bureaucratic barriers cost-effectively under certain conditions, thereby enhancing operational efficiency. According to their research, corruption may act as an efficient “lubricant” smoothing the means through rigid economic regulations and red tape, rather than solely hindering business; thus, it might not discourage cross-border M&As.

### 2.2. Equity strategies in cross-border M&As

The choice of equity strategy represents a critical component of decision-making in cross-border M&As. The significance of this strategy is evident as it directly affects both the immediate success of the acquisition and the post-acquisition performance of the acquirer [10]. An acquirer involved in cross-border M&As might choose a high-equity acquisition strategy to effectively manage and control the target’s tangible assets. In addition, pursuing high equity facilitates acquiring valuable organizational knowledge and other intangible assets, which finally enhances the acquirer’s core competitiveness and market standing [35].

Equity acquisition offers a pathway for enterprises to gain control over targets; however, the specific degree of equity sought can vary significantly. Not all acquirers pursue a high equity proportion, challenging assumptions about desires for complete ownership. Chen and Hennart [9] highlighted that information asymmetry amplifies risks in acquisitions, leading many acquirers to pursue lower equity stakes to reduce these risks. Their research suggested that MNEs, particularly those sensitive to investment risks from information asymmetry, often find joint ventures more appealing than full acquisitions. Similarly, Chen [36] found that MNEs opt for joint ventures to minimize the high costs and risks commonly associated with cross-border M&As, finally facilitating a more efficient and effective international market entry. Developing equity strategies for cross-border M&As also requires a thorough understanding of external factors, such as the host country’s economic development level and its institutional environment [12], including corruption.

### 2.3. Corruption and equity strategies

The relationship between corruption and equity strategies in cross-border M&As remains somewhat neglected, with relatively limited literature work having been published [13]. One of the earliest studies addressing this issue was conducted by Smarzynska and Wei [14], who focused on international investment activity in Eastern Bloc countries. Their findings demonstrated that high corruption in the host country resulted in a preference for joint ventures rather than full acquisition. The authors supported this outcome by arguing that lower ownership levels reduce commitment and risks. Therefore, this strategy represented an economically rational approach to the extremely uncertain and unpredictable economic conditions in the Eastern Bloc region during the 1990s.

Building on institutional theory, Uhlenbruck et al. [16] hypothesized that firms respond to corruption pressures through the selection of joint ventures. They viewed partnerships as an adaptive mechanism for entering markets with corruption. Similarly, Tekin-Koru’s [37] research indicated that companies from less corrupt countries exhibit a greater tendency towards full acquisition than their counterparts from countries with equal or greater corruption; whereas, Asiedu and Esfahani [38] determined no significant correlation between corruption and the ownership decisions made by US MNEs. A different pattern was identified by Di Guardo et al. [13]; they observed a U-shaped relationship connecting host country corruption levels with the probability of firms choosing full control in cross-border M&As.

These studies offer valuable perspectives; however, their main focus remains on the corruption level in the host country alone. A critical factor influencing acquisition strategies in cross-border M&As is actually the difference in corruption levels between home and host countries. The concept of “corruption distance,” signifying this significant gap, therefore represents a crucial variable that merits inclusion in analyses of M&A strategies [5,37]. This study seeks to fill the identified gaps by assessing how corruption distance affects equity strategies, offering a new contribution to the research field. In addition, this study evaluates potential variations in this relationship under differing conditions, with a specific focus on Chinese acquirers.

### 2.4. Corruption distance and equity strategies

This study argues that acquirers tend to favor high-equity acquisition strategies when the target firm operates in a country with a significant corruption distance. A high-equity strategy enables the acquirer to minimize transaction costs while reducing operational risks in an unfamiliar, potentially corrupt business environment [21]. By internalizing the target company through a high-equity acquisition, the acquirer not only gains significant control over the target’s operations but also obtains access to valuable intangible assets and tacit knowledge [13]. Such access holds particular value if the target resides in a highly corrupt nation as it offers the acquirer practical understanding of local management practices, thereby supporting the acquirer’s operational achievement.

A large corruption distance that favors the host country, indicating a significantly lower level of corruption compared to the home country, presents a more favorable and secure business environment for the acquirer [39]. Characterized by stable and transparent governance, a reliable and efficient public administration, and a robust legal system, such an environment safeguards the rights and assets of MNEs, cultivating operational efficiency [40]. Therefore, in such low-corruption environments, MNEs are more likely to pursue high-equity acquisitions to fully leverage the managerial benefits afforded by a transparent, accountable, and corruption-free business environment [13].

In contrast, in countries where corruption is pervasive and institutionalized, it significantly affects corporate governance practices. To effectively acquire local management skills and institutional intelligence in such an environment, MNEs tend to employ high-equity acquisition strategies [41]. Internalizing the target by acquiring a controlling interest grants these corporations privileged entry into the target’s resource base and knowledge reserves [42]. Accordingly, this process facilitates a swift understanding of the operational paradigms prevalent in the host country. Based on the above analysis, this study proposes Hypothesis 1:

***Hypothesis 1.*** *The corruption distance between the home country and the host country positively impacts the equity percentage of acquisitions in cross-border M&As.*

### 2.5. Moderating role of inherent political connections

Government ownership, representing a direct connection between enterprises and the state, can be understood as a societal resource [25] State-owned enterprises (SOEs), particularly those with strong ties to the government, often leverage this connection for international operational advantages, capitalizing on ownership benefits derived from these political connections [43,44]. Such benefits amount to favorable loan conditions, tax reductions, and lessened government scrutiny, factors which can all smooth the path for cross-border M&A activities [45]. However, state ownership a is also frequently accompanied by more complex management structures and the possibility of principal-agent conflicts [46]. Accordingly, SOEs typically exhibit lower operational efficiency when compared with privately owned firms. Moreover, SOE leadership, generally appointed by the government, might display high level of risk aversion. These executives could exhibit reluctance towards pursuing acquisitions involving high equity stakes when confronting increased uncertainty, for instance, operating in countries with high corruption. Instead, they might choose more conservative entry methods. The objective of this cautious strategy is to limit potential risks and preserve the enterprise’s stability in unfamiliar and unpredictable foreign markets.

SOEs engaged in cross-border M&A encounter further drawbacks from their outsider position, beyond the factors previously mentioned. Frequently, host countries perceive SOEs as extensions of their home governments, thereby raising doubts about their underlying motives and legitimacy. Intentions behind investments might be interpreted as politically driven instead of being solely focused on profit. Significant increases in the costs associated with cross-border M&A can result from this information asymmetry. Besides, while SOEs might receive government financial backing, host countries often interpret such aid as an inequitable market advantage. This perception of government meddling undermines the acquiring SOE’s legitimacy and obstructs the M&A transaction itself [47]. Therefore, the unique equity characteristics of SOEs, combined with their global image, present potentially significant barriers to achieving successful equity acquisitions in cross-border M&As. This situation could weaken the positive association between corruption distance and equity ownership levels. Based on the above analysis, this study proposes Hypothesis 2:

***Hypothesis 2.*** *Inherent political connections owned by acquirers weaken the positive effect of corruption distance on the equity percentage of acquisitions in cross-border M&As.*

### 2.6. Moderating role of acquired political connections

Executive political connections, conceived as an indirect connection between enterprises and governments, constitute a social resource accrued throughout a company’s lifespan [48]. Despite significant academic interest in the effect of such connections on business operations and performance, extant literature primarily centers on their implications for domestic business strategies, with comparatively fewer studies analyzing their relationship with multinational activities. Existing research suggests that executives cultivating political connections may enjoy facilitated access to financing during periods of financial distress, as they are more likely to benefit from government assistance and loan support. Empirical work by Khwaja and Mian [49], for instance, demonstrated that politically affiliated firms in Pakistan obtained loans from state banks that were 45% larger compared to loans secured by their non-politically connected counterparts. In addition, close political connections can afford executives privileged insights into government policy, enabling agile adaptation to regulatory shifts [50]. In cross-border M&As, this politically-derived information asymmetry empowers firms to anticipate government actions and formulate more proactive acquisition strategies. Therefore, political connections cultivated by executives in Chinese firms amplify the positive effect of corruption distance on equity acquisitions in M&A, as such connections facilitate access to both the requisite funds and information for M&A activities, thereby increasing the proportion of equity acquired.

Compared with inherent political connections such as those associated with state ownership, connections developed by individual executives typically encounter less governmental interference. These individually obtained political connections are less affected by governmental policy objectives during cross-border M&As. It allows decision-making processes that align primarily with the firm’s own interests. Firms utilizing executive-level political connections maintain a more subtle political posture, in comparison to SOEs which display an easily recognizable “political identity” in host countries. It subtlety renders them less prone to encountering resistance arising from host-country government sensitivities. These firms then face less rigorous analysis and fewer institutional challenges from host governments during cross-border M&As. It also lowers transaction expenses in multinational operations and motivates firms to adopt more assertive cross-border M&A approaches, thus strengthening the positive effect of corruption distance on equity acquisitions. Based on the above analysis, this study proposes Hypothesis 3:

***Hypothesis 3.*** *Acquired political connections owned by acquirers strengthen the positive effect of corruption distance on the equity percentage of acquisitions in cross-border M&As.*

**Fig 1:**
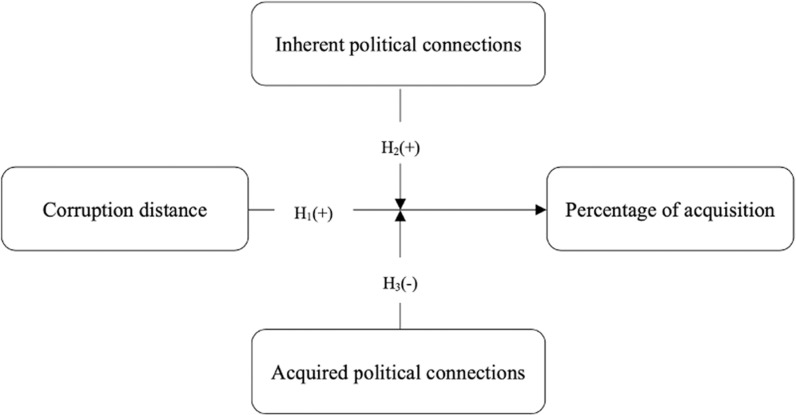
Research model.

The research model of this study is shown in Fig 1.

## 3. Method

### 3.1. Research setting

Several factors render China an optimal setting for conducting this study. First, Chinese firms have increasingly participated in cross-border M&As under the Chinese government’s ‘Go Global’ initiative [51]. Supporting this, the World Investment Report 2023 documented Chinese outward foreign direct investment (OFDI) reaching $147.85 billion, demonstrating the significant role these firms now play in international dealmaking. Secondly, China represents a transitional economy. Its institutional environment thus consists of substantive institutional voids alongside regulatory uncertainties that contribute to various forms of corporate corruption in cross-border M&A activities. Such a situation establishes a significant corruption distance between Chinese firms and their foreign counterparts [5]. Thirdly, the prevalence of political connections is high in China that significantly affect firm behavior, particularly where corporate governance systems are fragile [23]. Political connections therefore play a consequential role in corporate actions, which includes cross-border M&As [52].

### 3.2. Data collection

This study assesses cross-border M&A undertaken by listed Chinese companies between 2016 and 2021. This study commenced data collection using the Thomson SDC and Zephyr Global M&A Transactions Analysis Databases, aiming to identify cross-border M&A transactions involving a Chinese acquirer and a non-Chinese target. For the acquirers, specific exclusions were applied; companies based in Hong Kong, Macau, and Taiwan were removed. This decision reflects their unique operational environments under “one country, two systems” (Hong Kong and Macau) or unique legal structures (Taiwan), which lead to regulatory and corporate governance differences compared to mainland China [53]. In addition, for the targets, this study excluded those based in the British Virgin Islands and the Cayman Islands since these regions are frequently utilized for tax optimization, regulatory arbitrage, and holding structures rather than substantive operational M&As. This initial sample was then optimized to meet the following criteria: (1) exclusion of related party transactions; (2) exclusion of listed companies classified as ST or PT; (3) exclusion of companies with incomplete data; (4) exclusion of companies operating in the financial and real estate industries; their unique asset-liability structures, industry-specific reporting methods, and regulatory frameworks could introduce confounding factors into the analysis [54]. CSMAR was the source for data concerning corruption distance and other relevant financial details for the Chinese acquirers.

The final dataset comprises 393 completed cross-border M&A transactions executed by Chinese enterprises. The geographic distribution of targets indicates a concentration in Hong Kong (25.9%), followed by the United States (24.4%), Australia (5.1%), Japan (3.8%), and Germany (3.8%). The sample comprises acquisitions across 48 different host countries, such as Canada, Singapore, South Korea, the United Kingdom, and France. Sectoral analysis demonstrates a predominance of transactions in the manufacturing sector (238 deals, 60.6%), with the remaining 155 transactions (39.4%) occurring in various non-manufacturing sectors.

### 3.3. Measurement

#### 3.3.1. Corruption distance.

This study utilizes corruption indices sourced from Transparency International’s Corruption Perceptions Index. This index specifically measures the perceived probability that public officials in a country might engage in bribery, receive improper payments, or participate in other corrupt practices. The index ranges from 0 (*highly corrupt*) to 10 (*very clean*), with higher values indicating lower perceived levels of corruption.

The independent variable, corruption distance, is measured in this study as the absolute difference between China’s corruption index and that of the target firm’s host country [17]. When this corruption distance is larger, it signifies greater differences in corruption levels between the two countries involved. A high corruption distance (e.g., China = 3, Host = 8), for instance, points to asymmetric institutional settings where one country exhibits strong anti-corruption norms while the other has weak ones; whereas, convergent norms are implied by a low corruption distance (e.g., China = 3, Host = 4). This specific measurement enables a more detailed analysis focusing on how the perceived disparity in corruption between home and host countries influences acquirer equity strategies in cross-border M&As, extending beyond simple classifications of “high” or “low” corruption.

#### 3.3.2. Equity strategies.

The dependent variable, cross-border M&A equity strategies, is measured as the percentage of equity acquired by the acquiring firm in the target firm post-transaction [55]. The variable employs a continuous scale ranging from 0.1% to 100%. Prior research often utilizes binary classifications (e.g., ‘full’ versus ‘partial’ acquisitions); however, such divisions result in an inherent arbitrariness [9]. This study, by adopting a continuous measure instead, avoids conflating heterogeneous partial acquisitions (e.g., 20% stakes compared to 80% stakes) and effectively captures finer variations in strategic choices.

From a theoretical standpoint, acquiring a higher equity percentage in the target firm translates to greater ownership and thus enhanced control rights. In this study’s sample, the average equity stake acquired stands at 65.4% (with a standard deviation of 34.1). This distribution highlights the common use of majority-control strategies among Chinese acquirers, likely reflecting a preference for operational integration as a way to reduce the challenges associated with foreignness.

#### 3.3.3. Political connections.

Two types of political connections are measured in this study. The percentage of SOE shares in an acquirer’s total equity measures inherent political connections. Acquired political connections, alternatively, are measured here utilizing indicators developed from Yang and Zhang’s [56] research. Value assignments in this indicator depend on the past or present positions that executives have occupied in government hierarchies.

#### 3.3.4. Control variables.

This study incorporates controls for several key variables recognized for their influence on cross-border M&A strategies, including the acquirer’s age, financial leverage, deal size, industry relatedness, and the acquirer’s industry classification. Specifically, acquirer age acts as a proxy for organizational experience and resource accumulation, which can impact risk tolerance and long-term strategic orientation in cross-border M&A decisions [57]. Therefore, acquirer age is controlled, measured by the number of years from the establishment of the Chinese acquirer to the year of observation. Financial leverage, defined as the ratio of total liabilities to total assets, is also controlled as it reflects the firm’s financing capabilities [58]. Narayan and Thenmozhi [59] found that larger deals often involve greater integration challenges; thus, deal size is included as a control variable, measured by the logarithm of the total acquisition amount. Acquisition relatedness potentially offers benefits from shared capabilities and knowledge spillovers, thereby reducing integration risks [60]. Thus, acquisition relatedness is controlled utilizing a binary code (1 if acquirer and target share the same industry, 0 otherwise). Finally, the acquirer’s industry is coded (1 for manufacturing, 0 otherwise) to account for the stronger support certain sectors receive in China [54].

### 3.4. Analytical strategy

To evaluate equity strategies in cross-border M&As, this study concentrates solely on completed M&A transactions, deliberately excluding incomplete cases. To reduce potential sample selection bias, this study utilizes the Heckman two-stage selection model [61]. Specifically, by using a proper instrument in the first stage, this study aims to obtain an Inverse Mills Ratio that captures the correlation between the selection process (i.e., M&A completion) and the outcome variable (i.e., equity strategy). The resultant Inverse Mills Ratio is then incorporated into the second stage of the model for adjusting against sample selection bias.

In the Heckman model’s first stage, completed cross-border M&A transactions are coded as 1, while uncompleted transactions receive a code of 0. A Logit model is utilized in this first stage to correct for sample selection bias, chosen for its suitability with binary outcomes—in this case, distinguishing completed (1) from uncompleted (0) M&A transactions. Applying Logit regression allows this study to derive the Inverse Mill’s Ratio (*λ*) as:


$$\lambda \left(X_{i}^{\prime }\beta \right)=\frac{exp(X_{i}^{\prime }\beta )}{1+exp(X_{i}^{\prime }\beta )}$$

where $X_{i}^{\prime }\beta$ represents the linear predictor obtained from the first-stage model. This ratio offers a statistical adjustment for sample selection bias; it measures the effect of unobserved factors affecting transaction completion, thereby ensuring more reliable coefficient estimates in the second stage.

Additionally, the natural logarithm of the acquirer’s total assets was chosen as an instrumental variable to isolate exogenous variation related to M&A completion. Two critical criteria are met by this variable. Firstly, the instrument significantly predicts M&A completion (β=0.908, p<0.001), consistent with established findings that larger acquirers often experience higher regulatory approval rates due to their perceived economic benefits. Secondly, the instrument does not directly influence the second-stage outcome (M&A equity strategy), as evidenced by its non-significant coefficient in that stage (β=−2.475, p=0.144), which confirms its validity solely as a selection instrument. Hence, this study utilizes the acquirer’s size as an instrumental variable in the first-stage model to determine the Inverse Mill’s Ratio. The computed Inverse Mill’s Ratio is then integrated into the second-stage Ordinary Least Squares (OLS) model, effectively correcting for potential sample selection bias.

## 4. Results

Descriptive statistics for the variables analyzed in this study are presented in Table 1; these consist of the mean, standard deviation, and the Pearson correlation coefficient matrix. VIFs were calculated, indicating values ranging from 1.03 to 2.59. Since all VIFs decline below the threshold of 10, the absence of multicollinearity among the variables is confirmed. A Heckman two-stage selection model was employed for testing the proposed hypotheses.

**Table 1:** Descriptive statistics and correlations.

Variables	1	2	3	4	5	6	7	8	9	10
1. Percentage of acquisition	1.000									
2. Corruption distance	0.101**	1.000								
3. Acquirer age	0.051	−0.040	1.000							
4. Financial leverage	0.121**	−0.087*	0.355***	1.000						
5. Deal size	0.193***	−0.050	0.287***	0.316***	1.000					
6. Industry relatedness	−0.037	−0.061	−0.037	0.031	0.067	1.000				
7. Acquirer industry	−0.069	−0.042	−0.145***	−0.234***	−0.113**	−0.050	1.000			
8. Acquirer size	0.039	−0.138***	0.486***	0.628***	0.464***	0.125***	−0.266***	1.000		
9. Inherent political connections	0.030	0.190***	0.228***	0.134***	0.011	−0.136***	0.339***	−0.147***	1.000	
10. Acquired political connections	0.094*	−0.021	0.091*	0.002	0.028	0.001	0.114**	0.140**	−0.011	1.000
**Mean**	65.396	33.034	9.862	0.459	4.222	0.561	0.611	22.814	0.172	1.149
**S.D.**	34.072	11.493	6.522	0.201	0.947	0.497	0.488	1.565	0.378	1.768

* *p* < 0.05; ** *p* < 0.01; *** *p* < 0.001 (two-tailed test).

The results of this analysis are presented in Table 2, where cross-border M&A equity is the dependent variable. A baseline is established by Model 2, which incorporates only control variables. Model 3 then includes both independent and control variables, representing the primary model for testing Hypothesis 1. Building upon Model 3, Models 4 and 5 sequentially introduce the moderating variables along with their interaction terms involving the independent variables. The analysis derives in Model 6, the fully specified model, which incorporates all variables to allow for a comprehensive analysis.

**Table 2:** Results of Heckman two-stage selection model.

Variables	The first stage	The second stage				
	Model 1	Model 2	Model 3	Model 4	Model 5	Model 6
Acquirer age	−0.044** (0.018)	−0.189 (0.287)	−0.180 (0.288)	−0.083 (0.278)	−0.192 (0.279)	−0.098 (0.272)
Financial leverage	−0.547 (0.604)	13.324 (9.830)	15.747 (9.830)	19.246** (9.666)	15.428 (9.710)	18.714* (9.571)
Deal size	−0.622*** (0.141)	5.346*** (2.038)	5.367*** (2.019)	5.597*** (1.941)	5.386*** (2.022)	5.685*** (1.951)
Industry relatedness	−0.153 (0.178)	−2.707 (3.501)	−2.111 (3.506)	−2.062 (3.420)	−1.651 (3.457)	−1.667 (3.381)
Acquirer industry	0.008 (0.189)	−2.043 (3.574)	−1.698 (3.545)	−1.865 (3.413)	−2.731 (3.496)	−2.699 (3.395)
Acquirer size	0.505*** (0.126)					
Corruption distance			0.324** (0.142)	0.378*** (0.147)	0.283** (0.142)	0.326** (0.150)
Inherent PC				−15.976*** (3.932)		−14.116*** (3.929)
Acquired PC					2.534** (1.037)	1.882* (1.045)
Corruption distance × Inherent PC				−2.281* (1.282)		−1.689 (1.240)
Corruption distance × Acquired PC					3.733*** (1.284)	3.297*** (1.283)
Constant	−6.447*** (2.482)	36.046*** (9.584)	22.342** (11.336)	22.254** (11.144)	18.159 (11.480)	19.277* (11.367)
Year dummies	yes	yes	yes	yes	yes	yes
N	434	393	393	393	393	393
R-squared	—	0.071**	0.081***	0.116***	0.111***	0.136***
Wald Chi-square	—	21.68**	28.16***	55.76***	50.18***	76.38***

* *p* < 0.10; ** *p* < 0.05; *** *p* < 0.01; Standard errors in parentheses.

In Model of Table 2, the regression coefficient of corruption distance (β=0.324, p<0.05) indicates that greater distance in corruption indices between the home and host countries are associated with a higher proportion of equity, thus offering empirical support for Hypothesis 1. This result aligns with prior research suggesting that acquirers might favor full acquisition over joint ventures when significant corruption level differences exist between home and host countries; this choice may retain operational control and reduce risks related to corruption [5]. Additionally, Model 4 demonstrates the interaction term combining corruption distance and state ownership possesses a negative and significant coefficient (β=−2.281, p<0.10). This suggests that the positive association between corruption distance and equity proportion weakens when the acquiring firm has inherent political connections, thereby lending support to Hypothesis 2; whereas, Model 5 indicates a highly significant positive coefficient (β=3.733, p<0.01) for the interaction term between corruption distance and executive political connections. It indicates that acquired political connections strengthen the relationship between corruption distance and equity proportion. These findings empirically verify Hypothesis 3. For the complete Model 6, the R-squared value is 0.136, demonstrating that the full set of independent variables explains approximately 13.6% of the variance associated with the dependent variable (percentage of acquisition).

**Fig 2:**
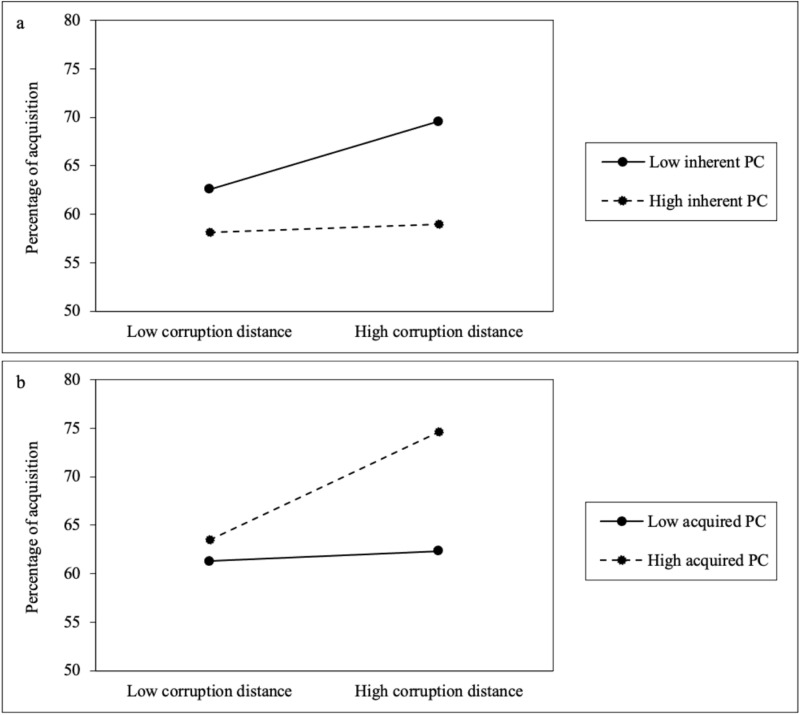
Moderating roles of inherent and acquired political connections.

Fig 2 represents the moderating effect of different types of political connections on cross-border M&As. As depicted in Fig 2a, the positive correlation between corruption distance and cross-border M&A equity is weakened in Chinese state-owned MNEs, characterized by their inherent political connections; whereas, Fig 2b demonstrates that this positive correlation is strengthened in Chinese MNEs where executives have cultivated acquired political connections.

Robustness tests 1 of the research hypotheses were conducted employing the Heckman probability selection model. Cross-border M&A equity was treated as the dependent variable and measured as a binary variable. Acquisitions exceeding 95% equity were classified as full acquisitions, denoted by a value of 1, while acquisitions with equity between 5% and 95% were classified as joint ventures, denoted by a value of 0. Table 3 presents the results of these robustness tests 1. The results from Model 3 exhibit that corruption distance exerts a significant, positive effect upon cross-border M&A equity (β=0.014, p<0.05). Further analysis in Models 4 and 5 indicates that this positive effect is weakened (β=−0.117, p<0.10) when inherent firm political connections exist. In contrast, when firms have acquired political connections, this positive effect is strengthened (β=0.218, p<0.01). Full Model 6 produced an R-squared value of 0.135, signifying that all independent variables explain approximately 13.5% of the variance in the dependent variable, the percentage of acquisition.

**Table 3:** Results of robustness tests 1.

Variables	The first stage	The second stage				
	Model 1	Model 2	Model 3	Model 4	Model 5	Model 6
Acquirer age	−0.041** (0.018)	−0.003 (0.011)	−0.003 (0.011)	0.003 (0.011)	−0.002 (0.011)	−0.098 (0.272)
Financial leverage	−0.563 (0.588)	0.206 (0.363)	0.309 (0.368)	0.550 (0.384)	0.320 (0.372)	0.533 (0.379)
Deal size	−0.582*** (0.137)	0.178** (0.081)	0.178** (0.081)	0.208** (0.087)	0.185** (0.083)	0.212** (0.086)
Industry relatedness	−0.124 (0.177)	−0.133 (0.128)	−0.110 (0.129)	−0.123 (0.134)	−0.078 (0.129)	−0.094 (0.132)
Acquirer industry	−0.068 (0.187)	0.169 (0.136)	0.185 (0.135)	0.173 (0.139)	0.156 (0.138)	0.141 (0.140)
Acquirer size	0.467*** (0.120)					
Corruption distance			0.014** (0.006)	0.015** (0.147)	0.136** (0.006)	0.014** (0.006)
Inherent PC				−1.005*** (0.204)		−0.960*** (3.929)
Acquired PC					0.055 (0.044)	0.029 (0.045)
Corruption distance × Inherent PC				−0.117* (0.061)		−0.093 (0.060)
Corruption distance × Acquired PC					0.218*** (0.075)	0.210*** (0.078)
Constant	−5.775** (2.361)	−1.399*** (0.394)	−2.006*** (0.468)	−2.104*** (0.502)	−2.209*** (0.496)	−2.204*** (0.509)
Year dummies	yes	yes	yes	yes	yes	yes
N	434	393	393	393	393	393
Pseudo R-squared	—	0.070***	0.077***	0.122***	0.096***	0.135***
Wald Chi-square	—	13.34	19.16*	47.83***	28.68***	57.71***

* *p* < 0.10; ** *p* < 0.05; *** *p* < 0.01; Standard errors in parentheses.

Moreover, this study addressed the potential for the corruption difference between China and host countries to be either positive or negative. Therefore, the sample was divided into two subsamples, acknowledging the relatively small number of cases in each. One subsample comprised cases where China’s corruption level was higher than the host country’s. The other subsample included cases where the host country exhibited higher corruption levels than China. Table 4 contains the second set of robustness results 2. For cases with a positive corruption difference, a positive effect of corruption distance on equity strategy was observed (β=0.471, p<0.05). Inherent political connections weaken this relationship (β=−2.428, p<0.05), while acquired political connections strengthen it (β=2.304, p<0.10). A similar positive effect on equity strategy was also identified for cases indicating a negative corruption difference (β=1.510, p<0.01). This effect was strengthened by acquired political connections (β=2.207, p<0.01). While inherent political connections seemed to weaken the effect in this subsample, the result lacked statistical significance (β=−7.879, n.s.). These supplementary tests, therefore, confirm the overall robustness of this study’s findings.

**Table 4:** Results of robustness tests 2.

Variables	Positive corruption difference		Negative corruption difference	
Acquirer age	−0.011	(0.282)	−0.140	(0.601)
Financial leverage	17.667*	(9.714)	3.687	(4.152)
Deal size	4.427**	(1.985)	3.021***	(0.385)
Industry relatedness	−0.902	(3.458)	−6.557	(13.370)
Acquirer industry	−3.472	(3.435)	4.122***	(0.984)
Corruption distance	0.471**	(0.186)	1.510***	(0.451)
Inherent PC	−13.855***	(4.053)	−19.114***	(6.802)
Acquired PC	2.033*	(1.057)	0.169	(1.795)
Corruption distance × Inherent PC	−2.428**	(1.172)	−7.879	(5.170)
Corruption distance × Acquired PC	2.304*	(1.270)	2.207***	(0.381)
Constant	18.068	(12.042)	11.409***	(2.745)
Year dummies	yes		yes	
N	369		24	
R-squared	0.126***		0.355*	
Wald Chi-square	71.92***		48.89***	

* *p* < 0.10; ** *p* < 0.05; *** *p* < 0.01; Standard errors in parentheses.

## 5. Conclusion

This study evaluates how the corruption distance between China and a host country influences the equity strategy choices of Chinese companies engaging in cross-border M&A from 2016 to 2021. Utilizing empirical analysis of a sample dataset, this study specifically evaluates the moderating role of the acquiring company’s political connections, both inherent and acquired. The findings indicate a strong correlation between a larger corruption distance and the likelihood of Chinese MNEs opting for high equity acquisition strategies. This pattern is attributed to the increasing transaction costs associated with establishing and preserving partnerships between Chinese companies and their overseas counterparts as the corruption distance widens. By securing a greater proportion of acquisition equity, Chinese acquirers can exert more control over the target company, facilitating internalization and thereby reducing transaction costs. In addition, this study indicates that inherent political connections in Chinese acquirers weaken the positive relationship between corruption distance and cross-border M&A equity levels; whereas, acquired political connections strengthen the positive effect of corruption distance on cross-border M&A equity.

### 5.1. Theoretical implications

Notwithstanding emerging market firms’ active participation in cross-border M&A, studies of these firms remain in an early stage [62]. This study significantly expands the body of knowledge on cross-border M&A by conducting a comprehensive analysis of MNEs from a key emerging economy—China. It is important to note that MNEs originating from emerging economies often exhibit diverse preferences compared to their counterparts in developed economies, largely due to their relatively constrained experience and expertise in outward direct investment [63]. Therefore, this study not only contributes valuable insights to the literature on cross-border M&A but also offers crucial theoretical grounding for understanding the strategic decisions by MNEs based in emerging economies.

Second, departing from prior studies [14] that solely concentrate on the effects of host country corruption levels, this study introduces the innovative concept of corruption distance—defined as the difference in corruption levels between the host and home countries. The aim of this study is to explore how corruption distance influences equity strategy decisions in cross-border M&A. Moreover, by analyzing the effect of corruption distance between China and host countries on cross-border M&A equity, this study addresses a significant gap in the extant literature concerning acquisition decisions, thereby enriching the research content in this domain.

Third, to appraise the moderating effect of corporate-government relationships in the acquisition process, this study distinguishes between two forms of political connections: inherent political connections (state ownership) and acquired political connections (executive political ties). The results suggest that Chinese acquirers characterized by inherent political connections may encounter legitimacy concerns and other obstacles during cross-border M&As, potentially reducing the positive association between corruption distance and equity acquisition; whereas, firms with acquired political connections demonstrate an amplification of this positive relationship. These findings offer a novel perspective through which we can better understand the complex role of political connections in affecting cross-border M&A activities.

### 5.2. Practical implications

It is necessary to approach corruption levels in both home and host countries, along with the corruption distance between them, with analytical depth for firms engaging in cross-border M&As, especially those from emerging markets. Acquirers might consider increasing the target’s equity acquisition ratio when this corruption distance is significant. Such a strategy offers potential benefits such as reduced transaction costs and enabling the acquirer to exert more significant effect over the target’s assets. China National Petroleum Corporation (CNPC) and Huawei represent examples of Chinese companies that have utilized this approach in specific acquisitions. Their objective was to secure greater control over strategic assets while enhancing operational influence in foreign markets.

Moreover, cultivating and nurturing strong political connections with the home government is vital for acquirers from emerging markets. Effective political connections with the home government become particularly crucial in environments marked by institutional voids that grant acquirers access to essential resources and enable them to address the informal rules governing business practices typical of cross-border M&As. ZTE Corporation offers a relevant example; the company has leveraged government support and connections specifically to reduce risks associated with its cross-border M&A activities.

Furthermore, SOEs from emerging markets face additional necessities. They should prioritize developing an ethical and responsible corporate culture. Building a trustworthy international reputation is also crucial, alongside enhancing corporate governance structures. Enhancements to governance could include implementing CSR initiatives and engaging in charitable donations. Carrying out these efforts is crucial for reducing the potential risks associated with political connections and finally ensuring long-term success in cross-border M&As.

### 5.3. Limitations and suggestions for future research

This study is not without certain limitations that require further extension from the presented findings. First, data on cross-border M&A transactions were sourced exclusively from listed companies for this research. The exclusion of non-listed firms and startup enterprises therefore represents a potential constraint on the generalizability of the results of this study. Future research incorporating a broader spectrum of international corporations could significantly enhance the robustness of these conclusions.

Second, the available data imposed restrictions that prevented the execution of certain robustness tests. Specifically, limitations in the datasets meant that robustness checks such as applying Canberra distance or employing logarithmic transformations for Euclidean distance were not feasible to implement. Integrating complementary methods for outlier detection in future work would allow for cross-validation of the current findings.

Third, considering that transaction cost theory and knowledge-based theory offer valuable perspectives on how the corruption distance can impact acquisitions, future analyses should determine whether the level of corruption in the home country surpasses or falls short of that in the host country. However, this study’s analysis was constrained by a limited sample size, as only 24 cases exhibited a higher corruption level in the host country relative to China, thus impeding a comprehensive subgroup analysis. Future studies could further explain this relationship.

Fourth, while this study provides a preliminary analysis of the different moderating roles of inherent and acquired political connections, future research could adopt a multidimensional perspective to further explore the moderating effects of political connections and other contextual variables. For instance, scholars could leverage network theory to distinguish formal political ties from informal ones [26]. Analyzing the differential consequences of these varying ties for decision-making in cross-border M&A would also be valuable. A more complete understanding of how firms strategically manage the challenges in cross-border M&A transactions would also benefit from incorporating institutional and cultural contextual elements, such as the opacity of regulations and norms governing business systems.

Finally, this study employed a cross-sectional design. Such a design captures relationships statically, specifically at the time deals are completed. Accordingly, the evolving dynamics between corruption distance and long-term post-acquisition performance might not be adequately reflected. Future analyses exploring the longitudinal consequences of corruption distance on the success or failure of M&As would therefore be highly valuable. Research of this nature might involve monitoring M&A performance over extended periods to evaluate how initial assessments regarding corruption distance impact eventual long-term outcomes.
